# Tissue-Resident Innate Immune Cell-Based Therapy: A Cornerstone of Immunotherapy Strategies for Cancer Treatment

**DOI:** 10.3389/fcell.2022.907572

**Published:** 2022-05-26

**Authors:** Rosalia Busà, Matteo Bulati, Ester Badami, Giovanni Zito, Daniela Claudia Maresca, Pier Giulio Conaldi, Giuseppe Ercolano, Angela Ianaro

**Affiliations:** ^1^ Research Department, Mediterranean Institute for Transplantation and Advanced Specialized Therapies (IRCCS ISMETT), Palermo, Italy; ^2^ Ri.MED Foundation, Palermo, Italy; ^3^ Department of Pharmacy, School of Medicine, University of Naples Federico II, Naples, Italy

**Keywords:** innate immune cells, macrophages, innate lymphoid cells (ILC), NK cells, tissue-resident immune cells, cancer, MDSC (myeloid-derived suppressor cell)

## Abstract

Cancer immunotherapy has led to impressive advances in cancer treatment. Unfortunately, in a high percentage of patients is difficult to consistently restore immune responses to eradicate established tumors. It is well accepted that adaptive immune cells, such as B lymphocytes, CD4^+^ helper T lymphocytes, and CD8^+^ cytotoxic T-lymphocytes (CTLs), are the most effective cells able to eliminate tumors. However, it has been recently reported that innate immune cells, including natural killer cells (NK), dendritic cells (DC), macrophages, myeloid-derived suppressor cells (MDSCs), and innate lymphoid cells (ILCs), represent important contributors to modulating the tumor microenvironment and shaping the adaptive tumor response. In fact, their role as a bridge to adaptive immunity, make them an attractive therapeutic target for cancer treatment. Here, we provide a comprehensive overview of the pleiotropic role of tissue-resident innate immune cells in different tumor contexts. In addition, we discuss how current and future therapeutic approaches targeting innate immune cells sustain the adaptive immune system in order to improve the efficacy of current tumor immunotherapies.

## 1 Introduction

Cancer is considered a major public health concern worldwide and is characterized by an uncontrolled division of altered cells. The human immune system recognizes tumor cells and induces a protective response to eliminate those cells. However, sustained tumors may protect themselves by developing immune escape mechanisms through multiple soluble and cellular mediators. In the last decades, the deep knowledge of tumorigenesis and the study of the complex interaction between the host and the immune system has been the goal for significant advances in anticancer therapy. Conventional anticancer therapy, such as surgical resection, radiotherapy, and cytotoxic drugs, involves multiple targeting of tumor cells. Though, the tumor tissue microenvironment can present a dysregulated, or weakened immune response which, in turn, uncovers pro-tumor activities favouring tumor expansion and progression (Dhara et al., 2022). Recently, new potential targets have been identified based on immunomodulatory therapies, with the aim to re-establish the host anti-tumoral immune response. Since the effect of cancer immunotherapy is largely dependent on the status of the immune system in the tumor microenvironment, the choice of therapy and the development of new therapies based on the immune status in the tumor microenvironment would be predicted to be effective (Sambi et al., 2019). Tissue-resident innate immune cells could be found in different human tissues, performing a strategic role at all stages of the immune response, from maintaining homeostasis to responding to infectious challenges to the resolution of inflammation to tissue repair and finally, to initiating antitumor response (Goff and Danforth, 2021). In humans studying immune cells and responses in tissues is challenging, due to the difficult accessibility of tissue-resident innate immune cells, the biggest pieces of knowledge concerning their responses in tissues have been obtained using murine models or studying immune cells drawn from blood (Segura et al., 2010; Gray and Farber, 2022). In recent years thanks to new knowledge obtained from these studies it has emerged that not only adaptive immune cells are the only effective cells able to eliminate tumors, but also innate cells are able to do it. Indeed, it emerged that natural killer cells (NK), dendritic cells (DC), macrophages, Myeloid-derived suppressor cells (MDSC) and innate lymphoid cells (ILCs), represent important contributors to modulating the tumor microenvironment and shaping the adaptive tumor response (Wang et al., 2019b). This review provides an overview of the different types of tissue-resident innate immune cells involved in the suppressor activity of anti-tumor immunity. The deep knowledge of the mechanisms underlying these processes could significantly improve the clinical utility of tissue-resident innate immune cells in cancer and eventually can support the identification of biomarkers for cancer prognosis and the development of novel therapeutic approaches for cancer treatment.

### 1.1 Tissue-Resident Dendritic Cells in Tumor Immunity

Dendritic cells (DCs) represent a heterogeneous family of immune cells, consisting of various subgroups of specialized antigen-presenting cells, mainly involved in initiating and regulating innate and adaptive immune responses (Wculek et al., 2020). Together with macrophages and B cells, they are considered the three major professional antigen-presenting cells (APCs). DCs play a critical role in promoting immunity by providing immunomodulatory signals, such as the secretion of cytokines and growth factors, but can also promote tolerance by presenting antigens to T cells (Patente et al., 2018; Wculek et al., 2020). They are a sort of sentinel able to collect a broad spectrum of environmental signals or stimuli such as bacterial and viral PAMPs and/or DAMPs, processing an extensive spectrum of specific tissue responses and influencing the immunological outcome, one of the most important, promoting T cell-mediated immunity (Levings et al., 2005; Steinman, 2006). DCs originate in bone marrow from unique precursor CD34^+^ that can differentiate into myeloid (MP) and lymphoid (LP) precursors. The first type of precursor gives rise to monocytes and DC precursors (MDP), which are further differentiated in common DC precursors (CDP), from which finally arise preclassical DC (pre-cDC) and plasmacytoid DC (pDC). In the last step of differentiation, pre-cDC will give rise to the most represented cDC subpopulations, named cDC1 and cDC2. Regarding the second differentiation way, or else LP, the ontogenic pathway is not completely elucidated, so nowadays we only know that it can give rise to pDC (Geissmann et al., 2010). DCs can be found in practically all tissue, they are a very plastic and dynamic cell population that can change its phenotype based on the tissue microenvironment in which is located. DCs represent the link between innate and adaptive immune responses, without inflammatory stimuli they are in an immature or tolerogenic state contributing to immune tolerance. Immature DCs express low levels of costimulatory molecules such as CD40, CD80, and CD86, besides they can infiltrate the tumor micro-environment performing a preponderant role in beginning antitumor immune response (Ganguly et al., 2013). The biggest knowledge concerning DC subpopulations have been obtained from the studies on murine DCs, mostly due to the wide range of tissue accessibility; unfortunately, the same type of characterization cannot be done for human DCs (Segura et al., 2010). Indeed, almost all studies on human DCs were performed mainly on peripheral blood where, among others, DCs constitute a rare cell population. Initially, DCs have been simply divided according to the cell localization, dividing them into resident lymphoid tissue DCs and migratory non-lymphoid tissue DCs (Haniffa et al., 2013). Nowadays, the development of new technologies, especially single-cell RNAseq, allowed to bring light new characteristics of this very heterogeneous cell population providing more information usable for classification criteria, including phenotypical, functional, and developmental criteria (Heath and Carbone, 2013).

Currently, DCs are divided into at least four wide groups using either functional or phenotypical characteristics. From a phenotypical point of view, all human DCs show a high expression of major histocompatibility complex (MHC) class II molecules (MHC-II) and of CD11c, which are expressed also on other cells, and many other molecules which allow their classification into various subtypes. Conversely, they lack key markers of T cells, B cells, natural killer (NK) cells, granulocytes, and monocytes. DCs subset can be classified into Conventional DC Type 1 (cDC1), Conventional DC Type 2, Plasmacytoid DC (pDC), and Monocyte-derived DCs (moDC), and each subset plays a different role within tumors and during their therapy (Kim et al., 2021).

The cDC1 are characterized by the presence of specific markers surface including thrombin receptor THRM (CD141), the chemokine receptor XCR1, C-type lectin CLEC9A and the cell adhesion molecule CADM1 (homologous of CD8α/CD103/XCR1 in mice) (Reynolds and Haniffa, 2015). The two mainly transcription factors involved in their generation are BATF3, a basic leucine Zipper ATF-Like Transcription Factor 3 and the IFN-regulatory factor 8 (IRF8). cDC1 are localized especially in peripheral blood and in lymphoid and non-lymphoid tissue, where they are specialized in cross-presentation, realizing the priming of CD8^+^ T cells against extracellular antigens such as bacteria and viruses (Haniffa et al., 2012). Recently, using different tumor murine models emerged that cDC1 also play a critical role in the induction of the cancer-immune cycle, exercised through the transport of antigens from tumor towards draining lymph nodes, inducing a robust activation/proliferation of CD8^+^ T cells or transfer of antigen to resident myeloid cells (Roberts et al., 2016; Salmon et al., 2016; Gardner et al., 2020). The antitumor immune responses mediated by cDC1s are critical in the mechanism of tumor rejection and responses to immunotherapies, like the immune-checkpoint blockade and adoptive T cell therapy (Kim et al., 2021). In addition, the presence of cDC1 within human melanoma tumors correlated with improved response to anti-PD-1 therapy (Barry et al., 2018) as well as with higher CD8^+^ T cell infiltration into tumors (Binnewies et al., 2019) which is associated with a positive prognosis across multiple tumor types (Fridman et al., 2012). In addition to PD-1/PD-L1 expression, it was also observed clustered expression of TIM-3 on cDC cells within tumors, particularly CD103^+^ cDCs. de Mingo Pulido et al. (2018), have shown that the use of αTIM-3 antibody-induced an increase in cell death within tumors and an improvement in response to chemotherapy, suggesting a key role of TIM-3 as a target for therapy. Recently, Roberts et al. (2016) have shown that anti-tumour activity of migratory cDC1s subtype can be done through the expression of chemokine receptor CCR7; in fact, in mice with cDC1 defective for CCR7, it was observed a loss priming of T cell in lymph nodes area and a lack of antigen hand-off to resident myeloid cells, which led to a failure of immune control with consequential increased tumor growth. Moreover, an analysis of tumour-infiltrating cell populations, isolated by human melanoma biopsies, showed that only CD141^+^ DC expressed a detectable CCR7 on the surface, demonstrating that CCR7 is particularly prominent on CD141^+^ DC in human tumors. In addition, it has been demonstrated that cross-presentation activity by cDC1 is improved by I interferon (IFN) signaling, and cells that lack IFNAR1 (IFN-α/β receptor 1) are unable to perform tumor-specific T cell priming and tumor elimination (Diamond et al., 2011; Fuertes et al., 2011).

The cDC2 are a heterogeneous subset of cells that co-express high levels of CD1c and SIRPα (CD172a) (homologous of CD11b and CD172a in mice) and a range of other markers that are tissue-specific (Shortman and Liu, 2002; Reynolds and Haniffa, 2015; Wculek et al., 2020). The transcriptional factors involved in cDC2 maturation and differentiation are mainly three, ID2 (Inhibitor of DNA binding 2), IRF8 (Interferon Regulatory Factor 8) and IRF4 (Interferon Regulatory Factor 4), which seems to have a preponderant role in CD8(+) dendritic cell differentiation (Rees et al., 1990; Li et al., 2016; Wculek et al., 2020). Using single-cell RNA-seq analysis, Villani and collaborators described two novel cDC1 subpopulations, namely cDC2 and cDC3 that show both the expression of CD11c^+^ but diverged for the expression of other molecular markers, including CD163/CD36 and CD32B (Villani et al., 2017). cDC2 are the dominant DC subset in blood but they are also localized in lymphoid and non-lymphoid tissue, which are involved in the induction of Th1, Th2, and Th17 responses (Haniffa et al., 2013; Haniffa et al., 2012; Segura et al., 2012). Moreover, recent studies in the literature suggest that cDC2 may be involved in presenting tumor-derived antigens to CD4^+^ T cells, which assist and support CD8^+^ T cells in their antitumor activity. Despite their role in tumorigenesis is still not well known, it emerged that they are effective stimulators of naïve T cell proliferation, required to mount an anti-tumor response (Villani et al., 2017). Besides, Binnewies and collaborators observed, in a murine model of melanoma, that the depletion of regulatory T cells into the tumoral site induced a cDC2 increase activity in eliciting intra-tumoral CD4^+^ T cell responses with subsequent tumor growth control (Villani et al., 2017). Similarly to the mouse model, it was observed an increase of CD4^+^ T in patients that show cDC2 abundance to the detriment of Treg suggests that the combination of high levels of cDC2 and low levels of Treg correlate with better tumor prognosis and with clinical responsiveness to immunotherapy (including anti-PD-1 therapy), though the increase of the levels of CD4^+^ T cell infiltration (Quezada et al., 2006; Balachandran et al., 2011; Wallin et al., 2016). It will be interesting to understand how the Treg may control cDC2 function and influence the anti-tumor CD4^+^ T cells response, both in melanoma and in other tumors (Sato et al., 2005; Binnewies et al., 2019).

Plasmacytoid DC (pDC) are characterized by the absence of CD11c and the expression of CD123 (IL-3R), CD303 (CLEC4C), and CD304 (neuropilin) markers (homologues to CD11c^int^ CD11b^−^ B220^+^ SiglecH^+^ CD317^+^ in mouse) (Dzionek et al., 2000; Collin et al., 2013). They arise in two different ways, directly from LP precursors and indirectly by CDP precursors, through the MP precursor’s line. The transcriptional factors that are essential for pDC development belong to the family of E2.2 expressed in both humans and mice (Liu, 2005; Cisse et al., 2008; Cheng et al., 2015). pDCs are a subset of DC specialized in response to viral RNA and DNA infection thus, for this reason, they express very high levels of TLR7 and TLR9, the two toll-like receptors specialized in signal transduction of viral and self-nucleic acids (Lande et al., 2007; Gilliet et al., 2008). The ligation of viral antigens to their TLR7 and TLR9 induces a very strong release of type I interferon (IFN-I) together with other inflammatory cytokines, including IL6 and TNFα (Patente et al., 2018). The role of pDCs in human tumors is less known compared to the other DCs subset and the data in the literature sometimes results controversial. The state of the art of pDCs asserts that they have an inert role in anti-tumor immune responses, but it also emerged that most cancers, including breast cancer (Sisirak et al., 2012), melanoma (Gerlini et al., 2007), and ovarian carcinoma (Labidi-Galy et al., 2011) are highly infiltrated by pDCs (Le Mercier et al., 2013; Kranz et al., 2016). Le Mercier et al. (2013) reported that pDCs infiltrating human primary tumors, represent an important prognostic factor associated with poor outcomes (Treilleux et al., 2004). Other studies reported that pDCs are able to limit tumoral progression, probably through IFN-α secretion (Kranz et al., 2016). Additionally, in tumor sites, it has been identified tumor-associated pDCs (TApDC) that, compared to normal pDCS, express a partially mature phenotype and an altered IFN-α, ΤΝF-α, and IL-6 production, able to induce an increase in Treg expansion, are associated with tumor progression with a poor overall prognosis (Hartmann et al., 2003; Treilleux et al., 2004; Labidi-Galy et al., 2011). Conversely, it was observed, *in vivo*, that an intra-tumoral injection of a TLR7 ligand led to TApDC activation displaying a potent curative effect, suggesting that TApDC could become an efficient therapeutic target (Kranz et al., 2016). Thus, several therapeutic protocols have been developed in cancers to stimulate pDC production of IFN-α. Among these, Imiquimod, a TLR7 agonist, has notably been used in cancer therapy because of its antitumoral action associated with the activation of NF-κB, which leads to the induction of proinflammatory cytokines such as IFN-α (Schon and Schon, 2008).

Monocyte-derived DCs (moDC) are a particular subset of DCs that differentiate separately from CD14^hi^ monocytes in humans (homologous to Ly6C^+^ in mice), in response to inflammation conditions (Jordan et al., 2020; Wculek et al., 2020). The knowledge acquired in the last years on human DCs was obtained using differentiated monocytes by *in vitro* culture with GM-CSF and IL4 (Sallusto and Lanzavecchia, 2018). The main surface marker of MoDCs overlaps with those expressed in macrophages, cDC2s and other immune cells, including CD1c, CD11b, CD14, CD209, CD172a CD1a, and CCR2 (Guilliams et al., 2014; Wculek et al., 2020). Recently, the evaluation of the Fc receptors FcγRI and FcεRI expression on moDCs allowed a better distinction between subsets, as moDCs express high levels of activating Fc receptors for IgG (FcγRs). Even if the physiological relevance of MoDC is unclear, it was noted that moDCs are produced in response to inflammation-inducing IFN-γ by CD4^+^ T cells promoting Th1 immune response. Phenotypical and functional alterations in moDCs have been identified in patients with different types of cancer, including breast cancer (Ramos et al., 2012) chronic lymphocytic leukemia (Toniolo et al., 2016), chronic myeloid leukemia (Brown et al., 2014), colorectal cancer (Orsini et al., 2013) and cervical neoplasia (Lopes et al., 2017). The most observed phenotypical alteration was reduced levels of specific markers involved in antigen presentation and lymphocytes activation, including HLA-DR, CD80, CD86, and CD83. Besides, phenotypical alterations were related to loss of function in inducing proliferation of both CD4^+^ and CD8^+^ T cells (Toniolo et al., 2016). The peculiarity of the mo-DCs to present the antigens in both MHC class I and class II molecules have been extensively used in the clinic, mostly as vaccines to induce anti-tumor immune responses in cancer patients. In the last decade, the use of DCs is considered a hopeful adjuvant for inducing immunity to cancer and their manipulation could represent a great potential for cancer immunotherapy (Thurner et al., 1999). A series of clinical trials on cancer therapy aimed to promote DCs activation, and consequently T cell priming against tumor antigen through the administration of specific cytokines and or adjuvant, such as FLT3L, GM-CSF and/or agents blocking a series of soluble factors released by cancer cells or specific signaling pathways that contrast DCs maturation (Saito et al., 2008; Merad et al., 2013; Johnson et al., 2018; Kerdidani et al., 2019).

### 1.2 Tissue-Resident Macrophages in Tumor Immunity

Tissue resident macrophages (TRMs) represent an important cell component of the innate immune system, with a wide distribution in every tissue throughout the body (Epelman et al., 2014b). Despite macrophage’s origin being thought to be derived from circulating blood monocytes infiltrating the tissue and differentiating into macrophages, recent literature has demonstrated that macrophage ontology is not that simple (Locati et al., 2020). Several recent pieces of evidence showed that TRMs can have embryonic progenitors, such as liver resident Kupffer cells (KCs), lung alveolar, microglia, splenic, and peritoneal macrophages (Hashimoto et al., 2013; Yona et al., 2013). Interestingly, these cells are fully differentiated before birth and self-renew in a monocytes-independent manner. However, some classes of TRMs, such as adult cardiac and skeletal muscle, derive from yolk-sac, and foetal monocytes progenitors, thus they can be substituted by blood monocytes (Epelman et al., 2014a; Wang et al., 2020). TRMs have a key role in innate immunity, as they represent the first line of defence that our body put in place upon infection with pathogens or microbes. In addition, they function by presenting antigens to T cells thus stimulating T cell response in a different types of disease conditions (Greenhalgh et al., 2018). Furthermore, macrophages maintain tissue homeostasis, by specifically contributing to the clearance of cellular debris (Herzog et al., 2019), tissue repair and remodelling (Bosurgi et al., 2017). In order to exert their functions, macrophages get activated by different stimuli coming from the tissue microenvironment *in vivo* and *in vitro* by a specific cocktail of cytokines. For a better comprehension of macrophages phenotype, Mills and collaborators classified them as classically activated TRMs (M1) and alternatively activated ones (M2) (Mills et al., 2000). While M1 macrophages release proinflammatory cytokines (TNF-α, IL-1β, IL-6, among others) and produce reactive oxygen species (ROS) to promote inflammation and defend against external pathogens, M2 ones stimulate the secretion of IL-10 and TGF-β to inhibit inflammation and to promote tissue repair and angiogenesis. However, a defined difference between these two classes of macrophages cannot be done, as their polarization can be switched according to the tissue conditions, whether it is in a steady-state or a pathological state. Thus, despite the efforts recently done to characterize M1 and M2 macrophages, a real definition of how TRMs work in physiological conditions is far from being achieved. Given their role within organs, TRMs have an important role during tumorigenesis, as they interact directly with tumor cells during progression and metastasis. Together with monocytes-derived macrophages, they represent the most abundant cells within a tumor, representing an important component of the tumor microenvironment (TME). It has been proposed that almost 50% of the tumor mass is generally represented by tumour-associated macrophages (TAMs) (Poh and Ernst, 2018). Thus, TAMs can have then different origins, although they all exert similar functions even if with different molecular mechanisms. For instance, it has been shown in transgenic mouse models of lung adenocarcinoma that while monocyte-derived macrophages stimulate tumor dissemination, TRMs are sufficient to induce tumor growth (Loyher et al., 2018). Similar results were obtained in TRMs-depleted transgenic mouse model (Csf1^op/op^), showing that alveolar macrophages depletion did not affect the dissemination of mammary tumor cells (Qian et al., 2009). However, this is not the case of pancreatic ductal adenocarcinoma (PDAC), where the pro-tumoral role of TRMs and monocyte-derived macrophages seems to be the exact opposite (Zhu et al., 2017c). These observations clearly suggest that the contribution of each macrophage population to tumor progression is strictly correlated with the tissue they reside. On the same lines of evidence, even the response to chemotherapy is different between TRMs and monocyte-derived macrophages, as it might change according to the type of tumor. Indeed, Loyher et al. (2018) elegantly demonstrated that monocyte-derived macrophages recovered faster from cyclophosphamide treatment when compared with TRMs in a transgenic mouse model of lung cancer. TAMs, either monocyte-derived macrophages or TRMs, are associated with tumor progression and poor prognosis, because of their role in controlling tumor survival and resistance to conventional therapies. In particular, different studies support the hypothesis that tumor-promoting macrophages have a M2-like phenotype (Mantovani et al., 2002; Boutilier and Elsawa, 2021), while the M1-like one is associated with anti-tumor properties (Noy and Pollard, 2014; Ubil et al., 2018; Wanderley et al., 2018). However, as discussed above for macrophages during homeostasis, macrophage polarization is influenced by cues and stimuli by the microenvironment (Sica and Mantovani, 2012; Najafi et al., 2019; Gunassekaran et al., 2021). For instance, different studies showed that TGF-β increased in hepatocellular carcinomas the expression of TIM-3, an immune checkpoint blockade inhibitor, thus leading to 1) M1 to M2 macrophages polarization, and 2) increased tumor progression and metastatization (Yan et al., 2015). This is a very important aspect, as the induction of an M1-like polarization could be an important therapeutic strategy to generate tumor-suppressive macrophages. TAMs have a pivotal role in defining therapeutic efficiency (Zitvogel et al., 2008; Hughes et al., 2015). For instance, they inhibit T cell response *via* inhibition of cytotoxic CD8^+^ T cells, either by expressing inhibitory immune checkpoint molecules (PD-L1 and PD-L2), blocking antigen presentation or by the secretion of immunosuppressive proteins, such as IL-10, TGF-β, and prostaglandin E_2_ (Blumenthal et al., 2001; Munn and Mellor, 2003; Matlack et al., 2006). In addition, they modulate T cell exclusion from the tumor by the activation of MMPs and cathepsins, and by stimulation of fibrotic mechanisms (Nielsen et al., 2016; Zhu et al., 2017c; Quaranta et al., 2018). Interestingly, it has been shown that when TAMs are depleted, cytotoxic CD8^+^ T cells increased their presence in the tumor context, thus improving the therapeutic outcome of the treatments (Denardo et al., 2009; Andreu et al., 2010). Along with inhibition of T cell response, TAMs are also responsible for chemotherapy and radiotherapy resistance and tumor relapse. Recent works demonstrated that standard care of treatment for several tumors determine a release of bioactive factors, such as VCAM1 and CCL2, that are involved in the increased macrophages infiltration in the tumor microenvironment (Kalbasi et al., 2017; Takahashi et al., 2020). In addition, *in vivo* studies on the prostate cancer model clearly showed that macrophages depletion further improve the docetaxel chemotherapy response by reducing tumor progression (Guan et al., 2019). Furthermore, macrophages induce resistance to chemotherapy by suppressing cancer cell apoptosis *via* release of soluble factors (colon and ovarian cancer model) (Feng et al., 2011; Au Yeung et al., 2016), or by exosomal delivery of miRNA-21 (gastric cancer cells) (Zheng et al., 2017). Finally, TAMs reduce immune checkpoint blockade therapy (ICB) *via* the expression of inhibitory immune checkpoint molecules (PD-L1, PD-L2, and TIM-3), thus blocking T cell response (Thommen et al., 2015; Anfray et al., 2019). For all these reasons, TAMs have been considered an important target in tumor immunity, although preclinical studies, as well as clinical trials, did not define in the past a proper clear-cut on possible therapies aiming at TAMs eradication. Thus, different approaches are currently understudies to target macrophages for anti-cancer therapies. Among others, therapies to deplete macrophages, inhibit monocyte-derived macrophages recruitment, and stimulate TAMs repolarization towards an M1 phenotype are currently under investigation in a preclinical stage, as well as in clinical trials. Macrophages depletion *via* CSF-1R blockade as monotherapy to affect tumor growth, despite the encouraging preliminary data in preclinical studies (Strachan et al., 2013; Quail et al., 2016), did not provide substantial benefits for the treatment of established solid tumors (O'Brien et al., 2021). On the contrary, a combination of tumor resection followed by macrophage depletion did provide a valuable reduction of melanoma recurrence and metastasis (Tham et al., 2015). Thus, combined therapies, along with the identification of the right timing of the treatment itself, might be the path to contrast tumor progression. Different approaches have been described to deplete macrophages, either in normal or tumoral tissues. For instance, liposomes loaded with clodronate have been shown to reduce tumor growth in mouse models of mammary cancer. In addition, its combination with protein kinase inhibitors, such as sorafenib, was able to drastically diminish tumor angiogenesis and metastasis in a hepatocellular carcinoma model (Zhang et al., 2010). Interestingly, different groups successfully attempted to eradicate TAMs using trabectedin, a chemotherapic used for the treatment of ovarian cancer and sarcomas. Trabectedin acts by stimulating macrophages apoptosis *via* activation of TRAIL-R2, a death receptor specifically expressed in macrophages (Allavena et al., 2005; Germano et al., 2013; Gordon et al., 2016). Thus, trabectedin is currently under analysis in combined therapies in several clinical trials. TAMs depletion can be obtained also by inhibiting CSF1/CSF-1R signaling axis, *via* monoclonal antibodies or small molecule inhibitors. In addition, targeting macrophages’ surface receptors (CD52, CD206, FR-β, among others) is another approach that has been recently taken for the same purpose. In all cases, these attempts result quite encouraging in the preclinical set, and some of them are now in clinical trials in combined chemotherapies for the treatment of lymphomas and chronic lymphocytic leukemia (NCT00069238, NCT01361711, and NCT01030900). Blocking monocyte-derived macrophages recruitment in the tumor microenvironment is another approach that has been tried to target TAMs. This type of therapy mostly relies on monoclonal antibodies aiming at the inhibition of the interaction between monocyte chemokines and their specific receptors. The most studied signaling axis in this context has been the CCL2/CCR2, as CCR2 is highly expressed in tumors and have been shown to stimulate macrophages recruitment in the tumor microenvironment (Lim et al., 2016). Also, in this case though, while the pre-clinical studies were encouraging, the clinic ones did not provide important results for the treatment of some type of prostate cancers (Pienta et al., 2013). However, the combination with anti-PD-1 immune checkpoint blockade therapy demonstrated quite efficiency for metastasis inhibition of mouse models of bladder and lung cancer (Tu et al., 2020).

Finally, TAMs repolarization towards an M1-phenotype is another valid strategy that has been developed in recent years. Macrophage re-polarization has been obtained with different methods, including treatments with TLR agonists (van Dalen et al., 2018), CSF-1R (Pyonteck et al., 2013) and PI3Kγ (Kaneda et al., 2016) inhibition. TLR agonists, including LPS and several lipoproteins, determine the activation of the NFkB signaling pathway, thus stimulating the production of pro-inflammatory cytokines typical of the M1 phenotype. For instance, Poly:IC, a synthetic molecule mimicking viral dsRNA, binds to TLR3 and induces macrophages polarization and colon cancer arrest. However, TLR agonists result cytotoxic for use in anti-cancer therapies, thus stimulating alternative methods for their *in-situ* delivery. Interestingly, TLR7/8 agonists loaded into nanoparticles induced *in vitro* and *in vivo* polarization of M1-like macrophages in different models of solid tumors, including lung and colon adenocarcinoma (Rodell et al., 2018). CSF-1/CSF-1R axis inhibition, despite being firstly considered a valid strategy for TAM depletion, is now a well-accepted method to repolarize macrophages. An elegant work published in 2013 clearly demonstrated in a glioblastoma multiforme (GBM) tumor model that CSF-1 blockade did not eradicate TAMs, but instead “re-educate” them within the tumor microenvironment by decreasing M2 macrophage gene signature, and at the same time promoting overall survival in patients with GBM (Pyonteck et al., 2013). Furthermore, CSF-1 blockade was found to stimulate TAMs polarization and improved animal survival in mouse models of hepatocellular carcinoma and PDAC (Zhu et al., 2014; Ao et al., 2017). Finally, PI3Kγ selective small molecules inhibitors nicely demonstrated their effectiveness in polarizing TAMs in mouse models of PDAC, thus promoting CD8^+^ T cell infiltration and tumor arrest.

### 1.3 Tissue-Resident Myeloid-Derived Suppressor Cells in Tumor Immunity

Myeloid-derived suppressor cells (MDSCs) are a heterogeneous group of immature myeloid cells, derived from the bone marrow hematopoietic precursor cells, which constitute one of the main suppressive cell populations of the innate immune system (Veglia et al., 2018). In physiological conditions, immature myeloid cells differentiate into the different innate immune cells, such as macrophages, granulocytes, and dendritic cells, and migrate to the corresponding tissues, exerting their normal immune functions (Groth et al., 2019). In pathological conditions, such as infectious diseases, cancers or autoimmune disorders, deregulation on myeloid differentiation occurs, which, combined with a persistent stimulation of myelopoiesis, results in the expansion of MDSCs (Consonni et al., 2019; Lim et al., 2020). This deregulated generation and expansion of immunosuppressive MDSCs is promoted by a series of cytokines, such as GM-CSF, VEGF, IL-1β, IL-6, and IL-10 (Cheng et al., 2021). Significantly increased immature myeloid cells have been observed in the bone marrow and peripheral blood of patients with cancer (Cheng et al., 2021), and the presence of enriched MDSCs have been related to poor prognosis for multiple types of cancer (Jiang et al., 2015; Tian et al., 2015; De Cicco et al., 2020). In fact, throughout the entire pathological process that leads to tumor formation, MDSCs increase up to10-fold and migrate to the periphery, exerting their suppressor activity interfering with the normal functions of circulating T and other immune cells involved in the anti-tumor immunity (Safarzadeh et al., 2019; Nakamura and Smyth, 2020; Ma et al., 2022). Unlike mice’s MDSCs, where these cells have been well characterized, human MDSCs are less clearly defined. Typically, they are described as lineage cells that co-express high levels of CD33 and CD11b surface markers but lack HLA-DR. Human CD33^+^CD11b^+^HLA-DR^−^ MDSCs can also be subdivided in three distinct populations of CD14^+^CD15^−^ monocytic-MDSCs (M-MDSCs), CD15^+^CD66b^+^CD14^−^ granulocytic-MDSCs (G-MDSCs) and CD14^−^CD15^−^ early-MDSCs (E-MDSCs), which comprised more immature progenitors (Gabrilovich and Nagaraj, 2009; Mandruzzato et al., 2016; Ma et al., 2022) myeloid markers, such as PD-L1, CD40, CD49d, CD80, CD115, and CD124, which all mediate immunosuppression, has also been discovered to describe specific patterns of MDSCs (Gabrilovich and Nagaraj, 2009; Mandruzzato et al., 2016; Ma et al., 2022). Two functional proteins, such as CCAAT/enhancer-binding protein (c/EBPβ) and STAT3, which promote generation, differentiation, and expansion of MDSCs, despite are not surface markers, could help to define two different MDSCs subgroups (CD11b^+^HLA-DR^−^c/EBPβ^+^ and CD33^+^HLA-DR^-^STAT3^+^) and could provide new diagnostic and therapeutic tools for cancer immunotherapy (Lechner et al., 2011; Wu et al., 2011; Wang et al., 2019a). MDSCs have a key role in accelerating the progression of cancer, by producing a broad range of suppressive factors that prevent immune cells’ anti-tumor reactivity (Ma et al., 2022). The main mechanisms by which MDSCs act as immunosuppressive cells are oxidative stress, amino acid consumption, cytokines secretion, cell-cell interaction and exosomes release. MDSCs mediate immunosuppressive effects under oxidative stress, producing reactive oxygen species (ROS), nitric oxide (NO), and reactive nitrogen species (RNS). Increased ROS production induces damages on adaptive immune response by interfering with TCR CD3ζ expression, which acts on IFN-γ expression, hampering activation, viability and proliferation of T cells (Belikov et al., 2015; Ohl and Tenbrock, 2018; Cheng et al., 2021). MDSCs express nitrogen-oxygen synthase 2 (iNOS). The upregulated expression of iNOS leads to NO production that suppresses T cell’s function by inhibiting JAK3/STAT5 activation (Bingisser et al., 1998) and decreasing MHC class II expression (Harari and Liao, 2004). NO also induce T and NK cell apoptosis in tumor microenvironment through different mechanisms, such as impaired expression of the Bcl-2 family proteins, increased expression of the p53 tumor suppressor protein, damage of mitochondrial functions, DNA fragmentation, and activation of the caspase cascade (Umansky and Schirrmacher, 2001). Additionally, in tumor cell aggregation sites enriched with MDSCs, NO reacts with superoxide forming the RNS, namely peroxynitrite, a strong nitrifying agent. It can nitrate tyrosine residues in T cell receptor, inducing reduced IL-2 production with a consequent impaired T cell activation and proliferation (Bentz et al., 2000; Cobbs et al., 2003; Szabo et al., 2007; Gabrilovich et al., 2012; Cheng et al., 2021) (Feng et al., 2018). Besides, peroxynitrite can modify TCR conformational flexibility affecting its interaction with MHC class I molecules, causing the decreased response of cytotoxic CD8 T cells to antigen-specific stimulation (Nagaraj et al., 2007). Additionally, peroxynitrite prevents antitumor infiltration of antigen-specific CD8 T cells by nitration of CCL2 chemokine (Molon et al., 2011). The second MDSCs’ immunosuppressive mechanism consists in the exhaustion of some amino acids with a key role in T cell functioning. The high production of arginase-1 (Arg-1) by MDSCs causes L-arginine deficiency in the tumor microenvironment, which either provokes the cell cycle arrest to the G0–G1 phase of T cells (Rodriguez et al., 2007) and the downregulation of TCR expression, inducing T cell dysfunction and tumor escape *in vivo* (Rodriguez et al., 2002). Another essential amino acid for T cell activation is cysteine (Levring et al., 2015). Dendritic cells (DC) and macrophages can import extracellular cysteine and export it in the tumor microenvironment, making this amino acid available for T cell utilization. MDSCs competitively import extracellular cysteine, but, contrary to DC and macrophages, MDSCs are not able to export cysteine, due to the lacked expression of cysteine transporters, preventing T cell activation (Srivastava et al., 2010). The MDSCs overexpression of indoleamine 2, 3-dioxygenase (IDO), an enzyme that, metabolizing tryptophan, has been referred to support immunosuppressive properties of these cells. Tryptophan depletion causes T cell proliferation arrest (Srivastava et al., 2010) and antigen presentation impairment (Fallarino et al., 2006). Besides, highly IDO levels produced by MDSCs promotes the differentiation and expansion of T regulatory (Treg) cells, exacerbating the inhibition of anti-tumor T cells function (Curti et al., 2007). In response to tumor microenvironment, MDSCs acquire the ability to produce multiple immunosuppressive molecules, such as cytokines, chemokines, and growth factors (Lechner et al., 2010). The tumor microenvironment is characterized by high levels of IL-10 and MDSCs are the principal producers of this cytokine (Cheng et al., 2021) IL-10, in turn, strengthens the immunosuppressive ability of MDSCs in a vicious cycle, by upregulating the expression of different immunosuppressive molecules (Xiu et al., 2015; Lamichhane et al., 2017). IL-10 produced by MDSCs induce increased expression of lymphocyte activation gene 3 (LAG3) and the consequent decreased IL-2, IL-12, and IFNγ secretion by T cells, which hampered their proliferation and anti-tumor activity (Vuk-Pavlovic et al., 2010; Li et al., 2015). As IL-10, increased TGFβ production by MDSCs has been reported in various tumor types. TGFβ is a potent immune regulator cytokine that can inhibit proliferation, activation, differentiation, and cytotoxic activity of effector T cells (Cheng et al., 2021). This cytokine blocked Th1 differentiation and activation by silencing the expression of TBET and STAT4, which are key transcription factors for the formation of this important subset of anti-tumor T cells (Gorelik et al., 2002). Moreover, acting on Smad3 signaling, TGFβ could decrease IL-2 production (McKarns et al., 2004) and downregulated the expression of granzyme B and IFNγ (Zhang and Bevan, 2012). Highly production of IL-10 and TGFβ by MDSCs can also lead to the differentiation and expansion of Treg cells, by inducing FoxP3 and CD25 expression on naïve CD4 T cells (Fu et al., 2004; Heo et al., 2010). Besides, MDSCs can produce high levels of some chemokines, such as CCL3, CCL4, and CCL5, which drive CCR5-expressing Treg cells through the tumor microenvironment, supporting the tumor growth (Schlecker et al., 2012). MDSCs could also impair T cell trafficking in tumor-bearing hosts. ADAM17 (a disintegrin and metalloproteinase domain 17) expressed on MDSCs directly cleaves the ectodomain of L-Selectin on T cells to inhibit their homing to tumor sites and peripheral lymph nodes (Li et al., 2021). Other than suppressing anti-tumor immunity, MDSCs can directly promote tumor progression and metastasis by inducing stemness of tumor cells, angiogenesis, and degradation of extracellular matrix (ECM). Many immunosuppressive factors, such as IL-10, TGFβ, and IL-6 produced by MDSCs, are able to induce stem cell properties in various tumor cells (Schlegel et al., 2015; Zhu et al., 2017a; Yang et al., 2019). MDSCs produce high levels of VEGF (Shojaei et al., 2009), the most important cytokine involved in angiogenesis, which binding its receptor (VEGFR) on epithelial cells, promoting neo-angiogenesis by activating JAK2/STAT3 pathway. MDSCs also express high levels of VEGFR2, which, activated by VEGF secreted either by tumor cells or by themselves, lead to a vicious cycle that contributes to maintaining MDSCs angiogenic activity (Min et al., 2017). Besides, MDSCs produce matrix metalloproteinases (MMPs) that, degrading the ECM, contribute to tumor metastasis (Zhang et al., 2020). Another mechanism provided by MDSCs to suppress immune response is through cell-to-cell contact. MDSCs constitutively express on their surface molecules involved in the suppression of immune cells. Among these molecules, Fas ligand (Fas-L) is highly expressed by tumor-infiltrating MDSCs and can induce apoptosis of CD8 cytotoxic T cells by activating Fas-Fas-L axis, with a consequent local immune suppression as demonstrated in mice models (Zhu et al., 2017b; Rashid et al., 2021). Besides, MDSCs in tumor microenvironment bear high levels of ligands of negative immune checkpoint regulators, such as PD-L1 and Galectin-9, which respectively binding PD-1 and TIM3, inducing T cell anergy (Cheng et al., 2021). Moreover, it has been reported that MDSCs are able to induce decreased cytotoxicity, reduced IFNγ production and downregulated expression of NKG2D of NK cells, due to membrane-bound TGFβ in a cell-cell contact mode (Li et al., 2009). Additionally, through the cell-to-cell transfer of the metabolite methylglyoxal, MDSCs could paralyze T cells, reducing their anti-tumor activity (Baumann et al., 2020). Finally, MDSCs can also exert their immunosuppressive function by releasing exosomes. Similarly, to parental MDSCs, exosomes secreted from MDSCs contain pro-tumorigenic factors and can play a crucial role in immunosuppression, tumor growth, angiogenesis, invasion, and metastasis by distributing their contents into the tumor milieu. It has been demonstrated that MDSCs-derived exosomes contain matrix metalloproteinases (MMPs) and different cytokines, chemokines, and growth factors (CSF, VEGF, MCP, SDF1α, TNFα, and IFNγ), which establish a pro-metastatic microenvironment that allows the metastatic progression of tumor cells (Umansky et al., 2016). Moreover, MDSCs-derived exosomes can induce exhaustion and apoptosis of CD8 T cells by either increasing ROS production or inducing the activation of Fas/Fas-ligand pathway (Rashid et al., 2021). Additionally, MDSCs-derived exosomes bearing the membrane-bound PD-L1, could induce the transformation of naïve B cells into B regulatory cells, recently identified as an immunosuppressive cell population (Rosser and Mauri, 2015), thus inhibiting antitumor immune response (Lee-Chang et al., 2019). Finally, some microRNA contained in MDSCs-derived exosomes, such as miR-126a and miR9, promotes tumor angiogenesis by reprogramming endothelial cells (Baroni et al., 2016; Deng et al., 2017). The deep knowledge of the mechanisms by which MDSCs exert their powerful immunosuppressive functions and pro-tumoral activity could help to develop new effective immunotherapeutic strategies for the treatment of tumors or could intensify the effectiveness of tumor treatments already used. For these reasons, several clinical trials targeting MDSCs and their products are ongoing. Among these, the use of monoclonal antibodies (mAb) against immune checkpoints inhibitors seems to improve cancer patients’ outcomes, in combination with other anti-cancer therapies. Ipilimumab is a fully humanized mAb that acts blocking CTLA-4. Ipilimumab, alone or in combination with other anti-tumoral treatments, could potentiate the anti-tumor T cell response and could lower the frequency of MDSCs in tumor microenvironment, ameliorating the outcome of patients with different kinds of solid tumors (Hodi et al., 2010; Sade-Feldman et al., 2016; Tobin et al., 2018). Pembrolizumab, a PD-1 blocking mAb, alone or in combination with BL-8040, a CXCR4 antagonist, was approved to treat unresectable or metastatic solid tumors, due to its ability to reduce MDSCs number and increase effector T cell tumor infiltration (Redman et al., 2016; Vachhani and Chen, 2016; Syn et al., 2017; Bockorny et al., 2020). Another target of immunotherapies is the blockade of MDSCs’ recruitment into the tumor microenvironment, by the antagonist of some chemokine receptors highly expressed on these cells. A CCR2 antagonist, namely 747, displayed anti-cancer properties and potentiate the efficacy of sorafenib in a model of hepatocellular carcinoma (Yao et al., 2017). Other chemokine receptors antagonists, such as reparixin (anti-CXCR1/2), LY2510924 and ulocuplumab (anti-CXCR4), in association with chemotherapics agents, have shown significant results in the treatment of different solid tumors (Galsky et al., 2014; Schott et al., 2017; Ghobrial et al., 2020). Another important strategy for cancer treatment is the inhibition of MDSC activation, by inducing the transition of immature MDSCs in mature myeloid cells. All-trans retinoic acid (ATRA) and the active form of vitamin D have been recognized as ideal inducers of MDSCs differentiation and have been used for different types of both hematopoietic and solid tumors (Iclozan et al., 2013; Tobin et al., 2018; Fleet et al., 2020; Makitie et al., 2020). More recently, the use of CSF-1R inhibitors, such as GW-2580, Imatinib, and pexidartinib, due to their ability to inhibit the expansion of MDSCs, have revealed important results in different solid and hematopoietic cancer treatments (Giallongo et al., 2018; Edwards et al., 2019; Wesolowski et al., 2019). Finally, Arg-1 and iNOS, critical factors in MDSC-mediated immunosuppression, are the targets of inhibitor agents (INCB001158 for Arg-1 and L-NMMA for iNOS), which, in combination with immunotherapy or chemotherapy, have shown good results in some solid tumors treatment (Cheng et al., 2021; Chung et al., 2021).

### 1.4 Tissue-Resident Natural Killer and Natural Killer T Cells in Tumor Immunity

Natural Killer (NK) and Natural Killer T (NKT) cells are lymphocytes of the innate immune system playing pivotal roles in immune surveillance and response against virus-infected and tumor cells. NK and NKT cells share some common phenotypes and function such as the secretion of interleukin-2 (IL-2), interferon-γ (IFN-γ) and tumor necrosis factor α (TNFα) upon interaction with the ligand or antigen (Shimizu et al., 2020). However, they express distinct lineage development, tissue distribution, antigen recognition and regulatory mechanisms in health and cancer (Vivier et al., 2012).

#### 1.4.1 Natural Killer Cells

Human NK cells are commonly divided into two subsets: immune-regulatory cytokine-responsive CD56^bright^CD16^−^ and cytotoxic CD56^dim^CD16^+^ with potent IFN-γ, TNF-α, and GM-CSF secretion activity upon stimulation (Cooper et al., 2001). The diatribe of whether CD56^dim^ are a mature form of CD56^bright^ (Romagnani et al., 2007) or a distinct subpopulation originating from a separate lineage is still a matter of debate (Cichocki et al., 2019).

NK cells infiltrate has been found in several types of cancers (Mamessier et al., 2011; Ali et al., 2014). Interestingly CD56^bright^ more efficiently traffic to the TME as they respond to the chemokines produced within the tumor bed. For example, IFN-γ stimulates tumor-infiltrating immune cells to release CXCL9-11, which is known to recruit CD56^bright^ NK cells (Wendel et al., 2008). By contrast, highly cytotoxic CD56^dim^ usually express receptors for chemokines produced at low levels and their traffic within the tumor site is often insufficient (Berahovich et al., 2006). NK cells vary expression of chemokine receptors following cytokine stimulation and therefore the composition of the NK cells in the TME changes accordingly (Pachynski et al., 2012). For example, CD56^bright^ NK cells respond to IL-15 by upregulating the expression of the chemokine receptor CCR5 and induce migration to the TME. Conversely, the same cytokine inhibits infiltration of cytotoxic CD56^dim^ cells by dampening expression of CXCR4 and CX3CR1 receptors (Sechler et al., 2004). In patients with advanced melanoma, an inverse correlation between the abundance of circulating CD56^bright^ NK cells and patients’ survival was found, pointing out a role of these cells in the modulation of cancer response (de Jonge et al., 2019). Likewise, the proportion of CD56^bright^ cells and production of IFNγ have been reported to be significantly lower in patients with prostate cancer than in controls (Koo et al., 2013).

Recently, by single-cell profiling a population of CD56^bright^CD127^+^CD160^−^CD52^+^ cells, NK0 was identified from human bone marrow and represent the precursors of conventional NK2/CD56^bright^CD160^+^CD52^−^ cells and NK1/CD56^dim^Perforin^high^ cells (Crinier et al., 2021). In patients with acute myeloid leukemia, transcriptomic analysis of the bone marrow revealed inhibition of the NK cell effector function and, importantly, patients with a good prognosis exhibited increased levels in the population of CD160^+^ NK cells (Crinier et al., 2021). Differently to T cells, NK cells recognize their target with a mechanism known as “missing self,” where the specificity for a given antigen is dispensable. NK cell function is modulated by a dynamic balance of activating and inhibitory signals and adhesion receptors in response to “altered self” cells such as tumor cells. When the inhibitory receptors do not recognize their target *via* the interaction with “self-identifier” (HLA) molecules, NK cells kill their target (Elliott and Yokoyama, 2011). NK inhibitory receptors are killer cell Ig-like receptors (KIRs) which engage with HLA types A, B or C (Harel-Bellan et al., 1986; Marsh et al., 2003), and NKG2A which binds to the highly conserved HLA-E (Sivori et al., 1996; Islam et al., 2021). More lately, other inhibitory molecules have been added to the list such as CD161, KLRG1, SIGLEC7, SIGLEC9, PD-1, TIGIT, LAG3, and TIM3 (Cozar et al., 2021). Conversely, activation receptors include CD16, NKp30, NKp44, NKp46, NKG2D, NKG2C, and activating KIRs. NK-cell activity is also enhanced by co-receptors like DNAX accessory molecule 1 (DNAM1) (Sanchez-Correa et al., 2012), NKp80 and 2B4 (Freud et al., 2017). For many years, it has been believed that NK cells uniformly recirculate. However, alongside conventional NK (cNK) cells, NK cells resident in the peripheral tissues, termed tissue-resident NK (trNK) cells have been reported in liver, kidney, skin uterus, salivary glands, and adipose tissue (Sojka et al., 2014; Fan and Rudensky, 2016; Sun et al., 2019). These tissue-resident lymphocytes do not recirculate in the blood or lymphatic system and have a distinct phenotype, like for example, the tissue-resident EOMES^−^T-bet^+^CD49a^+^ NK cells in the human liver (Sun et al., 2019). NK cells present immune-regulatory functions as they promote the activation of other innate and adaptive immune cells both by releasing cytokines and chemokines, or through direct cell–cell contact (Vivier et al., 2011). NK cells shape adaptive immune responses through the cross-talk with other cells such as T cells, B cells, and dendritic cells (DCs) (Malmberg et al., 2017). By inducing maturation of DC, NK cells trigger T cells mediated response; by producing IFN-γ, NK cells promote Th1 polarization (Moretta et al., 2008). Additionally, it has been proposed that NK cells can increase the function of cytotoxic CD8^+^ T cells by suppressing their state of exhaustion (Zheng et al., 2016) and, importantly, NK cell play a fundamental role in checkpoint blockade therapy by exacerbating antitumor or antiviral function of CD8^+^ T cells (Zhang et al., 2018). NK cells rapidly kill newly arising tumors or metastases, but their anti-tumor potency is less efficient against established solid formations. This is the result of the many strategies developed by tumors to escape immune surveillance, accompanied by a scarce capacity of NK cell to infiltrate the tumor site, as they tend to accumulate at the margins (Platonova et al., 2011). The tumor microenvironment (TME) is hostile to immune cells. NK cell effector function is limited by mechanisms of defense such as hypoxia, where tumor cells release abundant H2O2, thus limiting infiltration of CD56^bright^ NK cells (Izawa et al., 2011; Terrén et al., 2019). Additionally, excessive production of the metabolic enzyme Indoleamine 2, 3-dioxygenase 1 (IDO1) causes immunosuppression and NK and T-cell (Pietra et al., 2012). Likewise, Prostaglandin-E2 (PGE2) produced by cancer cells decreases NK-cell cytotoxicity (Galland et al., 2017). Additionally, tumor cells directly inhibit the expression of NK cell markers such as NKp30, NKp44, NKp46, and NKG2D by releasing soluble factors such as TGFβ (Lee et al., 2004; Sconocchia et al., 2012; Close et al., 2020). NK cells show impaired cytotoxic function hampered by cancer cells and upregulation of inhibitory receptors like NKG2A (Mamessier et al., 2011). The TME is also populated by different cell types that orchestrate suppression of the anti-tumor immune response. These include stromal cells, regulatory T cells (Treg), fibroblasts and myeloid-derived suppressor cells (MDSC). Inhibition of CD8^+^ T and NK cells is exerted by cell-to-cell contact or *via* release of TGFβ and IL10 or production of nitric oxide (Ghiringhelli et al., 2005). The TME negatively influences NK cell immune response. The phenotype, metabolism and function of intra-tumor NK cells dynamically change during the different stages of tumor occurrence and progression (Liu et al., 2021). In the early stages of breast cancer development, NK cells have cytotoxic functions, which are lost at a later phase, thus promoting tumor progression. NK cells have impaired functions and are exhausted in advanced cancers and are characterized by increased glucose and lipid metabolism (Liu et al., 2021). Downregulation of cytotoxicity genes was reported in biopsies of chemotherapy-resistant breast cancer samples by microarray expression assay (Garcia-Chagollan et al., 2018). In gastric cancer, the overall phenotype of NK cells did not differ between the population of circulating and infiltrating NK cells. What was reported, however, was impaired function in tumor infiltrating NK cells, with reduced IFNγ, TNFα, and proliferation (Peng et al., 2017). Likewise, in HCC tissues, a population of tumor infiltrating CD11b^−^CD27^−^ double negative NK cells was found to be characterized by poor effector functions and cytokine release (Zhang et al., 2017). In the TME, prolonged exposure to NKG2D ligands is associated with a reduction NKG2D expression, which causes NK cell function impairment and evasion of immune surveillance (Thompson et al., 2017). For example, in ovarian cancer, a reduction of the membrane-bound MICA/B proteins has been underpinned as an immune escape strategy adopted by tumor cells. MICA/B molecules bind to the receptor NKG2D which is expressed by NK cells, but also γδ^+^ and CD8^+^ T cells (Xie et al., 2014). NKG2D engagement corresponds to the inhibition of NK cell cytotoxic function and tumor progression (Xie et al., 2014). The metalloproteinases ADAM 10 and 17 are expressed by tumor cells and are implicated in immune surveillance escape. These mediate the shedding of B7-H6, ligand of NKp30. Increases of the B7-H6 in its soluble form determine drop in NKp30 expression and loss of NK effector function in different cancers (Schlecker et al., 2014; Pesce et al., 2015; Semeraro et al., 2015; Mantovani et al., 2019). PD-1 is an exhaustion marker on both T cells and NK cells (Liu et al., 2017b; Pesce et al., 2017). Importantly, NK mediated tumor response can be restored by blocking the immune checkpoint PD-1/PD-L1 axes (Liu et al., 2017b). Additionally to PD1, co-expression of a second exhaustion marker like TIM-3 has been associated to impaired NK cell function in many cancers, with reduced release of Granzyme B and IFN-γ and inhibited cytotoxicity (Seo et al., 2017). An interesting immune checkpoint is represented by the inhibitory receptor NKG2A. Its ligand, HLA-E is expressed by many different tumor cells (Zhen et al., 2013; Sun et al., 2017). Blockade of NKG2A/HLA-E axes with the antibody Monalizumab, for example, has proofed to unlock both NK cell and T cells function and corroborate anti-tumor immunity (André et al., 2018). Several studies have tried to unravel the role of NK cells in tumor immunity highlighting discrepancies mainly based on the type of tumor (Rezaeifard et al., 2021). Divergent results on the rate of NK cells infiltration in solid tumors have highlighted the urge to find reliable markers to address this question. The NK Cells Receptor (NCR) NKp46 has been recently identified as a robust biomarker to quantify tumor-infiltrating NK cells (Cózar et al., 2021). Although NK cells seem to reach the tumor bed to a lesser extent than other lymphocytes such as CD4^+^ and CD8^+^ T cells, their presence within the tumor correlates with a higher survival rate, as reported in head and neck squamous cell carcinoma (Weil et al., 2017; Concha-Benavente et al., 2018), colorectal cancer (Sconocchia et al., 2014), prostate tumor (Izawa et al., 2011) in gastric and esophageal cancers (Lorenzo-Herrero et al., 2019) and metastatic melanoma (Cursons et al., 2019b). Patients with metastatic skin melanoma showed better survival rates if infiltrating NK cells were detected in the tumor biopsies (Cursons et al., 2019a). Moreover, increased numbers of NK cells were correlated with a better response to anti–PD-1 immunotherapy and with an accumulation of pro-active DCs with a protective anti-cancer role at the tumor site (Barry et al., 2018). By contrast, the opposite has also been reported with inverse correlation between the advance stages of cancer and NK cells infiltrating the tumor (Vgenopoulou et al., 2003) or accumulating into the lymph nodes draining the tumor (Rezaeifard et al., 2019). Recently, a subset of CD49a^+^Eomes^+^ NK cells with known proangiogenic function has been described to accumulate at the site of liver tumor. This population has impaired cytotoxic function and reduced TNF-α release, strongly suggesting a pro-oncogenic activity in HCC (Zecca et al., 2021). NK cell infiltrate has been described in metastases, solid tumors and lymph nodes draining the tumor (Gulubova et al., 2009; Ali et al., 2014; Carrega et al., 2014). However, the extent of NK cell infiltration in the tumor greatly depends on the nature of the tumor and its localization. Some organs are more easily reached by NK cells such as the lungs, liver and kidney, while the intestinal tract appear to be less permissive (Remark et al., 2013). That said, high infiltration rate does not solely correlate with good prognostic factors. Expression of specific activating markers on NK cell surface represent a biomarker for more accurate prognosis (Schleypen et al., 2006), as described for the HLA-E receptor NKG2A and the activating marker NKG2C in endometrial cancer (Versluis et al., 2017). Tumor infiltrating NK and T cells represent a therapeutic target to tackle tumor. NK cells express the marker CD161, regulated by the gene KLRB1. CD161 expression orchestrates NK cell cytotoxic function in several cancers (Cheng et al., 2022). A good prognosis rate is observed in those cancer patients where KLRB1 is highly upregulated as infiltration of immune cells at tumor site and sensitivity to chemotherapy is KLRB1-dependent in many cancer types (Cheng et al., 2022). Nectar Therapeutics is developing an engineered IL-2 cytokine for the treatment of solid tumors. NKTR-214 is designed to sustain growth and survival of specific cancer-killing T cells and NK ells that specifically recognize a tumor target by targeting tumor infiltrating cells lymphocytes by binding to the CD122 receptor expressed by effector CD8^+^ T cells and NK cells (Bentebibel et al., 2019). However, it has also been reported that this effect is still partial, since a portion of the patients enrolled in the study displayed a proliferation of T_reg_ cells (Sullivan, 2019).

The efficacy of this cytokine has been tested in clinical trial also in combination with checkpoint inhibitor anti-PD1 demonstrating efficacy and safety in the treatment of solid tumor like melanoma, renal cell carcinoma, and non-small cell lung cancer (Diab et al., 2020). In Soft Tissue Sarcoma (STS), compared to circulating cells, intra-tumoral NK and T cells have upregulated TIGIT, a marker of exhaustion. TIGIT+ lymphocytes are considered prognostic in STS and recently it has been proposed that TIGIT blockade may be a promising clinical strategy in STS (Judge et al., 2020). Similarly, in colon tumor biopsies, tumor-infiltrating NK cells have increased levels of TIGIT expression than circulating NK, suggesting an exhausted phenotype (Zhang et al., 2018). Finally, CD155 is the ligand of both activating receptor DNAM1 and inhibitory ligand TIGIT and is expressed in many types of cancer. DNAM1 engagement with CD155 is associated to inhibition of NK cell function (Nakai et al., 2010; Li et al., 2020). NK cells participate to response to tumors and represent potential targets for cancer immunotherapy. However, the TME negatively affects NK cell function, phenotype, survival, and rate of infiltration as a mechanism of escape. This inactivation can be however reverted by using immune checkpoints inhibitors specific, for instance, for NKG2A and TIGIT, already used in clinical trials (Galot et al., 2021). Alternatively, some studies have addressed mechanisms to increase NK cell infiltration at the tumor bed by neutralizing soluble factors that suppress NK cells function such as TGFβ, already entered in clinical trials also in combination with anti-PD1 (Dodagatta-Marri et al., 2019).

#### 1.4.2 Natural Killer T Cells

Unlike conventional T cells, NKT cells recognize lipid antigens in a CD1d-dependent or independent manner (Vivier et al., 2012). CD1d^+^ NKT cells are divided into Type I or Type II NKT cells. Type I NKT cells are also known as invariant-NKT (iNKT) cells and express the invariant Vα14Jα18 TCR in mouse or Vα24Jα18 in human. This TCR recognize α-Galactosydcerimide (α-GalCer) lipid antigen (Terabe and Berzofsky, 2008). Human iNKT cells develop within the thymus and can be subdivided into functional subsets based on their expression of CD4 and CD8 into CD4^+^ iNKT cells, CD8^+^ iNKT cells, and DN iNKT cells (Lee et al., 2002). The DN and CD8^+^ iNKT cells have increased IFN-γ secretion and cytotoxic function upon activation while CD4^+^ iNKT cells have a pronounced helper function with release of type 2 (Th2) cytokines, such as IL-4 and IL-13 (Lee et al., 2002). In common with NK cells, human NKT cells express markers such as 2B4, NKG2D, DNAM-1, CD94, and NKG2A (Shimizu et al., 2020).

Frequency and function of intratumor or circulating iNKT cells have been assumed to correlate with overall survival in several types of cancers (Yanagisawa et al., 2002; Fujii et al., 2003; Tachibana et al., 2005; Schneiders et al., 2012), thus implying a role for iNKT cells in tumor immune surveillance. In details, reduced iNKT-cell numbers correlated with poor overall survival in head and neck squamous cell carcinoma (Molling et al., 2007) and acute myeloid leukemia (Najera Chuc et al., 2012). Conversely, increased numbers of intratumor or circulating iNKT cells have been associated with improved prognosis in colon cancer, prostate cancer, hematologic malignancies, and neuroblastoma (Metelitsa et al., 2004; Tachibana et al., 2005; Shaulov et al., 2008).

Human iNKT cytotoxicity cells against target cells may occur *via* TCR-dependent or independent signaling. During immune-surveillance, activation of iNKT cells can occur indirectly by cross-presentation of tumor lipids by APCs (Wu et al., 2003). Sialylated glycolipids on tumor cell membranes may become targets for iNKT cells and during tumor progression might be modified, representing a mechanism of immune surveillance escape. Several types of tumors, such as melanoma, small-cell lung cancer (SCLC), sarcoma, and neuroblastoma, highly express some gangliosides in comparison with corresponding normal tissue (Lo et al., 2010). These gangliosides might activate both CD4^+^CD8^−^ iNKT and CD4^−^CD8^−^ iNKT cells to produce IL-4 (Wu et al., 2003) and, acting as NKT cell ligands, might be related to prognosis in some cancers.

Activation of iNKT cells can also occur directly, *via* presentation of self-lipids by CD1d-positive tumors. CD1d expression has been found on solid tumors, such as prostate cancer (Tahir et al., 2001; Nowak et al., 2010), renal cell carcinoma (Chong et al., 2015), breast cancer (Hix et al., 2011), and tumors of the nervous system (Dhodapkar et al., 2004; Liu et al., 2013). For instance, in myeloma patients the frequency of iNKT cells in PBMCs are inversely correlated with disease progression (Dhodapkar et al., 2003; Jiang et al., 2018). This is due to the fact that primary myeloma cells express CD1d (Dhodapkar et al., 2003); however, its expression decreases in advanced stage cancer cells (Spanoudakis et al., 2009). Furthermore, human iNKT cells have been reported to kill CD1d^+^ osteosarcoma cells, but not CD1d^−^ osteoblasts, confirming the CD1d restriction of iNKT cell–dependent cytotoxicity (Fallarini et al., 2012).

Interestingly, iNKT cells from cancer patients are not impaired; rather they are in an anergic state as they can be expanded and activated by stimulation with DCs loaded with α-GalCer, thus pointing out iNKT cells as potential target for anti-cancer therapy (Iyoda et al., 2018). In myeloid leukemia patients, for instance, purified PBMCs-derived iNKT cells are responsive to stimulation with α-GalCer/CD1d-tetramer with production of IFN-γ, TNF-α, IL-2, and IL-4 and cytotoxic against autologous leukemic cells, that are CD1d^+^ (Metelitsa et al., 2003).

iNKT cells have the capacity to alter the immune tumor microenvironment thus influencing the ability of the host to limit growth of cancer cells. iNKT cells thus represent a population to be harnessed for the development of anticancer clinical therapeutics. Indeed, the identification of strong cell agonists, such as α-GalCer and its analogues, has led to the production of synthetic lipids that have shown potential in cancer vaccination and treatment (McEwen-Smith et al., 2015). Some approaches include using nanovectors/nanoparticle-based delivery systems (Ghinnagow et al., 2017) or α-GalCer loaded exosomes (Liu et al., 2017a). Moreover, vaccination with DCs pulsed with α-Gal-Cer with the aim to expand and activate iNKT cells in human cancer patients is also being evaluated (Richter et al., 2013; Shimizu et al., 2013).

To conclude, successful therapeutic treatments should aim at increasing the rate of tumor infiltrating NK and iNKT cells and rescuing their effector function.

### 1.5 Tissue-Resident Innate Lymphoid Cells in Tumor Immunity

Although NK cells are defined as the prototypical innate lymphocyte population, over the past decade, innate lymphoid cells (ILCs) have expanded the definition of innate immune cells. ILCs are mainly located in barrier tissues including skin, intestine and lung and are involved in multiple physiological and pathophysiologic processes (Mjosberg and Spits, 2016). Human ILCs are identified as Lineage^−^, CD127^+^ cells since they lack the expression of classical lymphocyte surface markers, the recombination activating gene (RAG)-rearranged antigen receptors and other surface molecules whilst express the CD127 (also known as IL-7 receptor). The lineage markers for ILCs identification in human include: CD3, CD4, CD8, CD14, CD15, CD16, CD19, CD20, CD33, CD34, CD203c, and FcERI in order to exclude macrophages, dendritic cells, red blood cells, T, B, and NK cells (Trabanelli et al., 2018). Likewise, mouse ILCs are identified as Lin^−^, CD127^+^, CD90^+^ where the lineage mix consists of: CD3ε, CD5, CD8α, CD11c, CD11b, CD19, B220, FCRεI, TCRαβ, TCRγδ, DX5, and Ter119) (Gomez-Cadena et al., 2020). ILCs are defined as the innate counterpart of T lymphocytes subpopulations. In fact, similarly to Th1, Th2, and Th17 cells, ILCs are classified in ILC1, ILC2, and ILC3 cells mirroring the expression of transcription factors and the cytokine secretion of their adaptive counterpart (Ercolano et al., 2020b). In particular, ILC1s express T-bet and produce IFN-γ and TNF-α, ILC2s express GATA-3 and secrete IL-4, IL-5, and IL-13 and ILC3s express RORγt and secreting IL- 17A and/or IL-22. In the human peripheral circulation, ILC3s also comprise a population of progenitor cells (referred as ILCPs) able to differentiate into all ILC subsets and natural killer (NK) cells (Lim et al., 2017). In addition, has been recently reported that ILCPs, thanks to the expression of CD62L are able to migrate to the lymph node (Bar-Ephraim et al., 2019). As tissue-resident cells, ILCs establish close interaction with other cells in the tissues contributing to the first-line defense against different threats shaping both innate and adaptive immune response by producing their prototypic, subset specific cytokine sets. The impact of ILCs in different diseases including allergy, asthma, rheumatoid arthritis, and inflammatory bowel disease, has been widely described during the last years (Pasha et al., 2019; Wu and Shen, 2020; Ercolano et al., 2022; Trabanelli et al., 2022). Nevertheless, our group and others reported a controversial role of ILCs in cancer showing both pro and anti-tumor effects according to the tumor type and the consequent cytokines and other cells that constitute the tumor microenvironment (TME) (Ercolano et al., 2019; Ghaedi and Ohashi, 2020; Jacquelot et al., 2022). Given their ability to secrete IFN-γ and TNF-α and the expression of the natural cytotoxicity receptor (NCR) NKp46, ILC1s are often teamed to NK cells and difficult to discriminate in both human and mouse (Zhang and Huang, 2017). However, at steady state these two subsets can be distinguished according to the expression of the transcription factors Tbet and Eomes (Daussy et al., 2014; Klose et al., 2014). In addition, unlike NK cells, ILC1s show a weak cytotoxic capacity due to their poor ability to secrete granzyme B and perforin (Vivier et al., 2018). Nevertheless, recent findings based on single-cell RNA sequencing and flow cytometric analysis identified an ILC1 subset with cytotoxic ability in the liver (Di Censo et al., 2021; Friedrich et al., 2021; Chen et al., 2022). Different data demonstrated that these two populations differ in both their development and distribution. In fact, while ILC1s are predominantly tissue-resident cells, NK cells are found mostly in the bloodstream and within secondary lymphoid tissues (Gao et al., 2017). Nevertheless, has been demonstrated that, under pathological conditions, including tumor development and metastasis, NK cells can acquire an ILC1-like phenotype. In particular, in a murine model of fibrosarcoma and melanoma, has been shown that NK cells were converted in ILC1s limiting the NK cell–mediated tumor immunosurveillance (Gao et al., 2017). TGF-β resulted to be the principal mediator involved in this switching of NK phenotype suggesting that the use of antibodies targeting TGF-β and its receptors may offer a promising strategy for cancer immunotherapy inhibiting the conversion of NK cells into ILC1s. In line with these findings, Salome et al. (2019) described an uncommon population of ILC1-like cells population with cytotoxic properties which were impaired in human acute myeloid leukemia (AML) by TGF-β and AhR ligands. Together with TGF-β, different cytokines present in the TME can modulate both the pro- and anti-tumoral effect of ILC1s. For instance, an IL-15 rich environment promotes the generation of a tissue-resident ILC1-like cells, characterized by a powerful cytotoxic activity towards cancer cells as demonstrated by using a MMTV-PyMT (PyMT) mammary tumor model (Dadi et al., 2016). Likewise, high levels of IL-15 have been observed in human colorectal cancer (CRC) biopsies, as well as high expression of T-bet and IFN-γ supporting the anti-tumoral role of ILC1s in this context (Mlecnik et al., 2014). In human melanoma, our own group reported an increase in the frequency of ILC1s in both peripheral blood mononuclear cells (PBMC) and tumor-infiltrated lymph nodes (TILN) of melanoma patients compared to healthy donors. Nevertheless, ILC1s from melanoma patients were functionally impaired as demonstrated by the reduced secretion of IFN-γ. By dissecting the different mediators present in the TME, we focused on adenosine and indoleamine. In fact, it is widely known that these two mediators are highly expressed in cancer and play a key role in the progression of melanoma and other types of cancer (Vijayan et al., 2017; Jennings et al., 2021). In particular, we observed that these two mediators are involved in ILC1 exhaustion leading to a reduction in type-1 cytokine secretion (Ercolano et al., 2020a). This effect was reverted by using an adenosine receptors inhibitor showing new evidence to sustain blocking these immunosuppressive pathways in melanoma patients by targeting the innate lymphoid cells arm.

ILC2s are assigned as a primarily pro-tumorigenic subset given their ability to produce type-2 cytokines, such as IL-13 and IL-5, and other immunosuppressive mediators. Different studies reported a higher frequency of tumor-infiltrating ILC2 in gastric, breast and prostate cancer. Particularly, in a first work published by Jovanovic et al. (2014), has been reported an increase of ILC2-derived IL-13 *in vivo* by using the 4T1 syngeneic murine model. Next, Salimi et al. (2018), observed an increase of ILC2s and investigated on the expression of different activatory and inhibitory receptors in tumor-infiltrating ILCs in both human breast and gastric cancer. Similarly, Trabanelli et al. (2017), found an enrichment of ILC2s in prostate cancer patients which was correlated with tumor stage and myeloid derived suppressor cells (MDSCs) frequency. Increased numbers of ILC2 have been showed also in the tumors of gastric cancer patients infected with *Helicobacter pylori* (Li et al., 2017). Furthermore, very recently, has been reported a link between ILC2 and tuft cells (a rare population of epithelial cells present at the gastrointestinal and respiratory tract). In particular, the tuft cells/ILC2 axis seems to be involved in the development of gastric cancer as demonstrated by using a murine model of intestinal metaplasia. This result was also confirmed in tumor microarrays of intestinal-type gastric cancer patients showing a unique correlation for tuft cells, ILC2s and survival in intestinal-type gastric cancer. IL-25 and IL-13 play a key role in dictating the crosstalk between tuft cells and ILC2s, in fact, the treatment with either α-IL13 or α-IL25 neutralizing antibodies significantly restrained tumor growth which coincided with a reduced frequency of both tuft cells and ILC2s in mice (O’Keefe et al., 2022). The pro- and anti-tumoral role of ILC2s is frequently associated with the overexpression of various cytokines involved in their activation including IL-33, IL-25, and TSLP commonly defined as “alarmins” (Roan et al., 2019). Our group recently demonstrated that IL-33 is highly expressed in tissues as well as in the serum of CRC patients. The high presence of IL-33 didn’t affect the frequency of ILC2s but increased their activity in term of IL-13 and IL-5 production which in turn sustain CRC progression through the modulation of the epithelial-to-mesenchymal transition (EMT) phenomenon (Ercolano et al., 2021). Conversely, it has been recently reported that in pancreatic adenocarcinoma, IL-33 induces ILC2 expansion that was accompanied by enhanced intratumoral CD8^+^ T cells pushing tissue-specific tumor immunity (Moral et al., 2020). Interestingly, they also reported that tumor-infiltrated ILC2s express the inhibitory checkpoint receptor PD-1 hypothesizing that PD-1 blockade could further boost ILC2 activation to enhance anti-tumor efficacy. In line with these findings, (Jacquelot et al., 2021a) demonstrated that PD-1 blockade increased ILC2 and eosinophil recruitment and enhanced anti-tumor responses in melanoma context (Jacquelot et al., 2021b). These findings suggest the need to further dissect investigation on the broad array of checkpoint inhibitors as target for ILC2 in cancer immunotherapy.

The role of ILC3s in cancer is emerging as complex and highly dependent on the tumor type as also described for their adaptive counterpart (Bruchard and Ghiringhelli, 2019). In fact, similarly to Th17 cells, ILC3s exert both pro- and anti-tumor functions showing different phenotypes according to the tissue microenvironment. Although ILC3s are generally dependent on the transcription factor RORγt, there are more complex subsets that further subdivide ILC3s in NCR^+^ ILC3s and NCR− ILC3s depending on the expression of the natural cytotoxicity receptors (NCRs) NKp46 and NKp44 in both human and mice (Melo-Gonzalez and Hepworth, 2017). In addition, lymphoid-tissue inducer (LTi) cells are a further subclass of ILC3 closely related to NCR- ILC3s, involved in lymph nodes development (Spits et al., 2013). ILC3s have been associated with the pathogenesis and progression of different type of cancer. For instance, a profound reduction of ILC3 NCR^+^ cells were observed also in acute myeloid leukemia (AML) patients which was completely recovered after two cycles of treatment with standard chemotherapy (represented by daunorubicin/cytarabine) (Trabanelli et al., 2015). Conversely, Liu et al. (2019) demonstrated that NCR− ILC3s were the tissue-resident cells mainly present in the liver of hepatocellular carcinoma-bearing mice. In particular, NCR- ILC3s supported the early phase of tumour development by promoting the establishment of an IL-17-rich tumor microenvironment in response to IL-23 sustaining the differentiation of other IL-17-producing cells. These findings suggest that NCR- ILC3s, as well as IL-23, could be an interesting target to exploit for the prevention of hepatocellular carcinoma progression. Given the essential role of LTi cells in generating secondary lymphoid tissues during embryogenesis, it is possible to hypothesize an involvement of these cells in cancer context. In fact, has been demonstrated that an IL-12 enriched environment promotes the recruitment and proliferation of NKp46^+^ LTi cells, able to inhibit tumor growth and thwart the development of lung metastases in a murine model of cutaneous melanoma (Eisenring et al., 2010). Importantly, (Jacquelot et al., 2021b) recently reviewed that LTi cells are implicated in the development of tertiary lymphoid structures that are formed in inflamed tissues (including tumor) and are composed by different immune cells driving the immune response against tumor development and progression and improving the clinical outcome (Jacquelot et al., 2021a). Moreover, in subcutaneous melanoma, it has been showed that LTi cells exert a tumoricidal effect by increasing the expression levels of adhesion molecules in the tumor which in turn promote the recruitment of adaptive immune cells and the subsequent tumor control (Eisenring et al., 2010). The ability of ILCs to foster the recruitment of other immune cells has been recently described in bladder cancer settings. In particular, Vanoni et al. (2021) demonstrated that ILCPs (the ILC3 progenitor subset present in the peripheral blood) interact with endothelial cells inducing, on one hand, the expression of adhesion molecules on the surface of endothelial cells and acquiring and, on the other, an ILC3-like phenotype sharing some phenotypical markers with LTi cells. However, in human high-grade bladder carcinoma samples, ILCPs are barely detected and their ability to upregulate adhesion molecule expression on endothelial cells was impaired (Vanoni et al., 2021). These findings could describe one of the mechanisms through which tumour cells block the infiltration of immune cells into the tumour site highlighting a new attractive target to regulate the immune infiltration and establish an antitumor immune response.

## 2 Concluding Remarks

**FIGURE 1:**
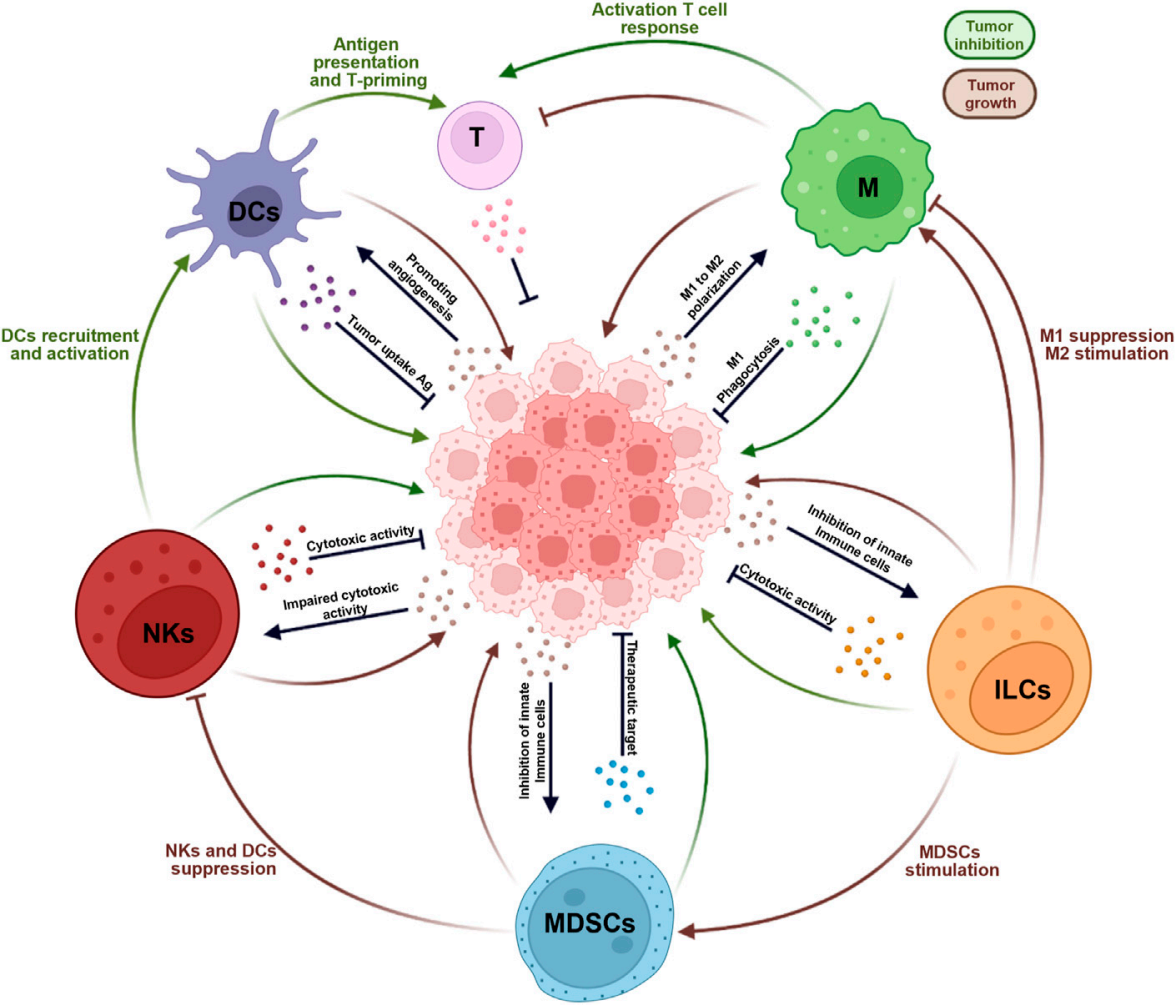
Orchestration of cancer immune control. Within the tumor each tissue-resident innate immune cells exert both positive (tumor inhibition, in green) and negative (tumor growth, in brown) immune regulatory functions. The tissue-resident innate immune cells are important contributors in modulating the tumor microenvironment though the secretion of growth factors, chemokines, and cytokines. As show in the figure, the mainly subpopulation involved are dendritic cells (DCs), macrophages, myeloid-derived suppressor cells (MDSCs), natural killer cells (NKs), and innate lymphoid cells (ILCs).

Immune system evasion is a distinctive hallmark of cancer (Hanahan, 2022). In the last decades, cancer immunotherapy has experienced important clinical progresses in the treatment of different types of cancer. For instance, immune checkpoint inhibitors such as ipilimumab, nivolumab, and pembrolizumab, whose primary purpose is to unleash effector T cells response, have recast the treatment of aggressive forms of tumor including melanoma, non-small cell lung carcinoma and colorectal cancer (Bagchi et al., 2021). However, cancer relapse and recurrence occur for most patients increasing the need to find new therapeutic targets to improve cancer immunotherapy. The tumor milieu is a complex assortment of immune cells, blood vessels, connective tissue cells and extracellular matrix molecules, which all exert a remarkable influence on the cancerous cells they surround. The better understanding of this microenvironment, with particular attention focused on the function of immune cells within this region is today of particular interest. In this context, tissue-resident innate immune cells exert both positive and negative immune regulatory functions (Figure 1), representing important contributors in modulating the tumor microenvironment and shaping the adaptive tumor response (Wang et al., 2019b). Thus, investigate on the innate-adaptive lymphocyte crosstalk in cancer, could represent an interesting approach in order to increase the efficacy of the current immunotherapies.
